# Levels and kinds of explanation: lessons from neuropsychiatry

**DOI:** 10.3389/fpsyg.2014.00373

**Published:** 2014-04-29

**Authors:** Sam Wilkinson

**Affiliations:** Department of Philosophy, Durham UniversityDurham, UK

**Keywords:** explanation in psychology, delusion, personal explanation, neuropsychiatry, levels of explanation

## Abstract

I use an example from neuropsychiatry, namely delusional misidentification, to show a distinction between levels of explanation and kinds of explanation. Building on a pragmatic view of explanation, different kinds of explanation arise because we have different kinds of explanatory concerns. One important kind of explanatory concern involves asking a certain kind of “why” question. Answering such questions provides a personal explanation, namely, renders intelligible the beliefs and actions of other persons. I use contrasting theories of delusional misidentification to highlight how different facts about the phenomenon that is being explained impose constraints on the availability of personal explanation.

## Introduction

Neuropsychiatry involves the study of people with mental illnesses in a way that makes use of the tools and understanding of the cognitive and brain sciences. As a result, a foundational question for neuropsychiatry is: What is the nature and extent of the contribution that the cognitive and brain sciences can make to our understanding of psychopathologies and mentally ill individuals? That is why, in this paper, I use neuropsychiatry to draw attention to two explanatory constraints. Although these constraints are important in all areas of the cognitive sciences, broadly construed, they are particularly visible in neuropsychiatry. One constraint concerns *levels* of explanation. The other constraint, which is often overlooked, often misunderstood, and is of particular importance to neuropsychiatry, concerns *kinds* of explanation.

I proceed as follows. I start by contrasting three general views about the nature of explanation, and opt for a pragmatic view. I then introduce and characterize both the “levels” and the “kinds” constraint within a pragmatic framework. I then illustrate the latter constraint by examining recent work on delusion. I end by addressing an illustrative objection.

## Three contrasting views of explanation

Before looking at explanatory constraints, it is important to reflect on what explanation is generally. Differing answers to the following two questions yield different views about the nature of explanation. These two questions are:

1. What kinds of things are the relata in explanations? (viz. When we say that x explains y, what kinds of things are the values for x and y? Or alternatively, what kinds of things are the *explanans* and the *explananda*.)
2. What is it for x to successfully explain y?

Following Faye (2007), I think it is useful to distinguish between three kinds of views of explanation, namely: *Formal-logical*, *Ontological*, and *Pragmatic* views of explanation. My aim is not to adjudicate between these, but rather to show that the pragmatic view provides an especially helpful way of approaching the issues in this paper.

### The formal-logical view

On the formal-logical view, first and famously put forward by Hempel (1965), an explanation is an abstract entity; in particular, it is a logically valid argument with propositional structure. Indeed, an *explanandum*, according to Hempel, is a proposition that follows *deductively* from an *explanans*. A number of things should be noted about this approach.

- Scientific and ordinary (everyday) explanations are profoundly different in nature. The things we call “explanations” in daily life never, or at best rarely, pick out logically related propositions.
- This characterization is prescriptive rather than descriptive. It is neither interested in capturing how we use the word “explain,” nor in capturing what scientists are actually engaged in doing when they explain things. It aims to tell us what something *ought* to be if it is to count as an explanation in this refined, ideal, sense. (One might alternatively put this in evaluative rather than constitutive terms and say that explanations are *good* explanations to the extent that they approximate this ideal.)
- Explanations are objectively “out there” to be discovered.

The formal-logical view of explanation includes a number of views of explanation besides Hempel's original covering-law version. For example, it includes Salmon's statistical-relevance model as well as the unificationist theory of scientific explanation as elaborated by Friedman (1974) and Kitcher (1989).

In answer to questions 1 and 2, sets of propositions explain other propositions, and they do so by standing in valid and sound deductive relations to each other. Practically speaking, although it may apply to areas of physics, it is too demanding to usefully apply to psychology, and it does not reflect what psychologists actually do or ought to do. Of course, given the aim of this view of explanation, that is not necessarily a criticism.

### The ontological view

On the ontological view, explanations are not made up of logically related propositions. They are made of concrete entities like, for example, objects, states of affairs, or events. For example, you might think that events explain other events. In particular, it is common within this approach, to think of causes explaining their effects. An instance of fire explains an instance of smoke.

So, in answer to questions 1 and 2 above, we get: Events (or states of affairs) explain other events (or states of affairs), and they do so by standing in predicable law-like causal relations.

A couple of things should be noted about this view:

- Again, scientific and ordinary (everyday) explanations are different in nature. We rarely explain things to each other by picking out law-like causal relations.
- Again, explanations are out there to be unearthed. You discover them. You find a particular event, and you unearth the explanation of that event, namely, its cause or causes.

One recent theorist, who buys into this account in philosophy of science generally, is Woodward (2003). Another, who applies a related view specifically to psychological explanation, is Donald Davidson. To simplify somewhat, Davidson (1970) takes causal *relata* to be not objects, not properties, but events, namely, he takes events to cause other events. He also takes explanations to require the picking out of a cause (which is an event) to explain an effect (which is also an event). However, these events are only explanatory “under a certain description.” In other words, he is sensitive to the fact that picking out events that are causally related is not sufficient to be explanatory: you have the pick them out in a *causally relevant* way. For example, to explain why the scales go down when weighing some plums, you appeal to the weight of the plums, not their color, even though those are two aspects of one and the same event (namely, the putting of purple plums on the scales). This has some affinities with the pragmatic view. However, we will see that, crucially, the pragmatic view opens up the possibility of non-causal explanation.

### The pragmatic view

According to a pragmatic view of explanation, an explanation is a good answer (and, we shall see, a variety of factors, both psychological and objective, may contribute to this “goodness”) to an explanation-demanding question. The relata of explanations are not events, nor are they propositions; they are speech acts that are heavily dependent on a number of contextual factors. The relevant contextual factors can include a number of things (for example, conversational context) but the most important for our purposes are the explanatory concerns of the demander of the explanation (which I will henceforth call “the demander”). An explanation has to address the explanatory concerns of the demander, and has to be (at least a candidate for being) considered satisfying. This potential subjective satisfaction is a necessary but not a sufficient condition of something being a good explanation. Obviously, there are many objectively bad explanations that we may wrongly consider satisfying (e.g., “just-so stories”). So they have to be satisfying in a non-illusory way. Different theories will flesh out what it is for something to be “satisfying in a non-illusory way,” but, very roughly, it will mean that it is true or accurate, which can then be cashed out in terms of corresponding to reality, or something weaker such as “usefulness,” or “assertability.” The finer details of this objective criterion are not as important for our purposes (viz. of distinguishing the pragmatic view and introducing explanatory constraints that are grounded in it) as the subjective criteria. These are that the demander has to understand the candidate explanation, and that the explanation has to address the demander's explanatory concerns.

Crucially, an explanation that is objectively good by the standards of either the ontological or formal-logical view, but which leaves the demander completely in the dark, is not considered a good explanation on the pragmatic view. Explanations are relative to a particular instance of a question being asked, and have to cater to the demander's epistemic state. The demander, it must be noted, is not necessarily an individual, but could be a collective. The “question” could be asked implicitly by the scientific community as a whole (or a subset of that community), or explicitly by an individual.

There are some varieties of the pragmatic view. The view was first introduced by Van Fraassen (1980). Achinstein (1983) has an attractive version that relies heavily on the tenets of ordinary language philosophy, and Faye (2007) puts forward his own refinements. Here is what all versions of the pragmatic view have in common, in particular, in contrast to the formal-logical and the ontological views characterized above.

- Scientific and ordinary explanation is essentially the same. The former simply has a more regimented context (viz. the explanatory concerns are regimented and shared across a community, namely the scientific community).
- Explanations, being the products of communicative acts, are not discovered as pre-formed entities. They are answerable to how things stand in the world, but they need to be selective and carefully formulated so as to be comprehensible to the demander of the explanation. In sharp contrast to both formal-logical and ontological views, explanations simply do not exist in a possible world devoid of inquiring beings that demand and give explanations. Furthermore, these explanations are demanded within a wider pragmatic context, whether it is everyday life, the court of law, the lab, or the clinic.

So, to sum up, in answer to questions 1 and 2, explanations are communicative speech acts, and they explain in virtue of satisfying the demander's explanatory concerns in a non-illusory manner (where “illusion” can arise at the level of truth or accuracy, or at the level of comprehension, namely, thinking that one comprehends when one does not). The epistemic or informational state of the demander of the explanation will in part determine her explanatory concerns, and her explanatory concerns will dictate the kind of explanation that would be satisfying. A broadly pragmatic view of explanation is what I will be building on for the rest of this paper.

### Different explanatory concerns about the same phenomenon

On a pragmatic view (in contrast to an ontological view), one phenomenon can arouse different explanatory concerns, each of which demand different explanations. Sometimes we have different explanatory concerns because we happen to be interested in different things. At other times, however, the phenomenon itself can impose constraints on what explanatory concerns are suitable, namely, what questions one should ask.

Suppose there is a plane crash. One can, for example, ask for an explanation of the plane crash in terms of poor decision-making, or neglected obligation. Or one may ask for an explanation in terms of technical problems with the plane; or in terms of the weather conditions. Depending on certain facts about the crash, either of these explanations may be unavailable. For example, if the weather had been so extreme that even the most skilled of pilots would have been unable to avoid a crash, then the explanation in terms of poor decision-making is unavailable. Conversely, if the pilot had been a terrorist who had deliberately crashed the aircraft, then an explanation in terms of the weather or the aircraft malfunctioning will be unavailable. Realizing the unavailability of certain explanations is extremely important since it should prompt us to not ask questions that have no answers.

The crucial point is this. Certain facts about the phenomenon that you are trying to understand can restrict what questions can be asked, what explanatory concerns you should have, what explanations will be available. Somebody looking to hold someone accountable for a plane crash in situations where the weather was too extreme, is asking the wrong question. This issue of asking the right questions is extremely important in all areas of science, and especially visible when looking at mental illness.

## Levels of explanation

The notion of “levels of explanation” is usually introduced without any particular commitment to a general view of explanation. However, it makes good sense to view it within a pragmatic framework. Indeed, the classic mention of “levels of explanation,” in David Marr's book *Vision* (1982), is phrased in terms of answering three kinds of *question*:

*Computational theory*: What is the goal of the computation, why is it appropriate, and what is the logic of the strategy by which it can be carried out?
*Representation and algorithm*: How can this computational theory be implemented? In particular, what is the representation for the input and output, and what is the algorithm for the transformation?
*Hardware implementation*: How can the representation and algorithm be realized physically? (p. 25)

Marr's three levels makes a point that applies to any talk of levels of explanation. It is the *functionalist* point of there being multiple realizability of high-level or functional properties in lower-level properties (e.g., the property of being a bottle-opener can be physically realized in a number of different ways, as long as it opens bottles). If our explanatory concerns are about the higher-level properties (e.g., computational properties) then addressing them by drawing attention to lower-level properties (e.g., hardware properties) will be unsuitable (nevertheless, lower level implementational properties clearly impose constraints on higher level properties: you cannot make a bottle opener out of cream cheese). If one has explanatory concerns that operate at a certain level, addressing them at a different level is at best, sub-optimal, and at worst, completely irrelevant or opaque.

Some theorists see what is called the “personal level” as just another level in this sense: as a particular functional level where we are talking about whole persons, what they believe, desire, feel etc. These “personal level” properties are (if we assume physicalism) physically implemented, but they could in principle be implemented by different physical states. Dennett's doctrine of the “intentional stance” seems to view things in this way. He presents us with the following thought experiment. Suppose:

“some beings of vastly superior intelligence—from Mars, let us say—were to descend upon us […] suppose, that is, that they did not need the intentional stance—or even the design stance—to predict our behavior in all its detail” (Dennett, 1981, p. 68).

The question then is: do these Martians miss out on anything in failing to use the intentional stance, the personal-level vocabulary of beliefs, desires etc.? According to Dennett, although they might be able to predict the exact motions of the fingers and the vibrations of vocal cords during an instance of a stockbroker buying shares in General Motors, if they fail to see

“that indefinitely many *different* patterns of finger motions and vocal cord vibrations—even the motions of indefinitely many different individuals—could have been substituted for the actual particulars without perturbing the market, then they would have failed to see a real pattern in the world they are observing” (1981, p. 69).

Note that even here, with its non-reductive take-home message, Dennett calls this “a *predictive* strategy.” The plan is to predict how a causal system will behave at the relevant fineness of grain. The finger motions are not a relevant fineness of grain for gaining a predictive understanding of the stock market. The intentional stance is the relevant fineness of grain for gaining a predictive understanding of persons.

If this is correct, then the distinction between levels and kinds of explanation that I want to put forward is unnecessary. There are only levels, and one level (perhaps the “top” level) is the “personal level.” Many theorists seem to subscribe to this view (which is somewhat encouraged by the presence of the word “level” in “personal level”). They take the challenge of connecting, say, a neurobiological story to a cognitive story to be the same kind of challenge as that of connecting a subpersonal and a personal story. I hope to show now that this is not the case.

## Kinds of explanation

The pragmatic view of explanation allows there to be as many kinds of explanation as there are kinds of explanatory concerns (or, which comes to the same thing, kinds of questions worth asking). And, crucially, we have explanatory concerns that are not just causal or mechanistic. We ask questions like, “Why did this person do that?,” “Why does this person believe that?” We trade on the fact that there are correct and incorrect answers to these questions, and that these answers genuinely inform us. However, they do not do so by giving us a causal, predictive, understanding of the situation. These questions demand personal explanations, and personal explanations are not merely another “level” of explanation, but a different *kind* of explanation.

We can illustrate the difference between the personal and subpersonal kinds of explanation, by reflecting on different kinds of explanation-demanding question. Roughly, whereas subpersonal explanation is mechanistic or causal, answering “How” and “How come” questions (respectively), personal explanation answers a “Why” question, where “Why” is understood in a certain way.

### Causal and mechanistic explanations

Within a pragmatic view one can distinguish causal and mechanistic explanation in the following way. A causal explanation merely tells you what causes what, whereas a mechanistic explanations is far more informative in telling how a certain phenomenon comes about. A causal explanation provides some degree of understanding, and a sufficient degree of understanding for some situations, for example, if one wants to avoid a particular effect. Thus we might establish that smoking causes cancer. We might not know exactly how it does so, but knowing that, at least, is enough to suggest that (*ceteris paribus*), if we do not want cancer, we should not smoke. We can think of causal explanations as answering a certain kind of question, namely, a “How come?” question. “How come he got cancer?” This is often expressed with a causal use of “Why,” as in “Why did he get cancer?” This causal use of “why” is very different from a justificatory use that we are about to encounter.

A mechanistic explanation answers instead a “How?” question. It provides not just the cause, but the mechanism whereby a certain causal process operates. Using the cancer example, it is not enough to know that smoking causes cancer: what is required is a description of the mechanism, for example, in terms of carcinogenic disruption of genetic material through radiation given off by substances present in tobacco smoke. Mechanistic explanation provides a greater degree of understanding than merely citing causes. It is reasonable to think of mechanistic and causal explanations as being the same kind of explanation insofar as the kinds of concerns addressed are of the same kind; namely, of predicting how a brute causal system will behave in relevant counterfactual circumstances.

### Personal explanations

As I said, we do sometimes use “why,” when our explanatory concerns are causal or mechanistic, for example, when we ask “Why is there a hole is the ozone layer?” We mean, “By what cause or process is there a hole in the ozone layer?” We know it is not there for a *reason*. However, when we ask, “Why is there a STOP sign at the end of that road?” we are asking for a reason, a justification, a *rationale*, for its being there. Along with the distinction between justificatory and causal uses of “why” in the *question* (“Why is there a STOP sign there?/Why did you raise your hand?” vs. “Why did he get cancer?”) we have the distinction between justificatory and causal uses of “because” in the *answer* (“Because there tends to be fast-moving traffic in the main road/Because I wanted to ask a question” vs. “Because he smoked too heavily”). Answering such a “why” question involves citing a person's reasons or grounds for believing certain things and acting in certain ways (or the general, publicly agreed, reasons, not attributable to a specific person, as in the case with the STOP sign).

With beliefs and actions, we often ask questions of one another: “Why do you believe that?” “Why did you do that?” In doing this, we are asking a very particular kind of question, and one that requires a very particular kind of answer. This answer is commonly called a *rational* explanation (Davidson, 1963). However, “rational” has a categorical and evaluative sense. The opposite of “rational” in the categorical sense is “a-rational” (or “non-rational”), whereas the opposite of “rational” in the evaluative sense is “irrational.” The way “rational” is used here is categorical, not evaluative. As we are about to abundantly see, you can have rational explanations of *irrational* phenomena. Indeed, something that cannot be given a rational explanation cannot be irrational; it is merely *a*-rational. Consider a nervous tick. You cannot ask why (in the justificatory sense) someone with a nervous tick is behaving the way they are. And, clearly, you cannot evaluate their reasons as bad reasons if there are none. However, because of this ambiguity with the word “rational” I have made the terminological decision to use “personal explanation” rather than “rational explanation.”

If you ask me, “Why did you raise your hand?” and I answer, “Because I wanted to ask a question,” that is normally a satisfying explanation. If I tell you a full physiological story about what happened up until the point when my hand went up, that may be interesting, but it is not an answer to *that* question. You were after a *reason*. You wanted to know what I was hoping to achieve by raising my hand. The same applies when you ask the question “Why do you believe this?” You are after *reasons* for my belief, not any mechanistic story. You want to know what grounds I have, if any, for believing something.

### Is personal explanation causal explanation?

Now, you might agree that these are common and valid explanatory practices. However, you might question whether there is anything fundamentally different about them. Why are these not causal explanations? We know that certain beliefs and desires in certain contexts will give rise to certain actions. This seems rather causal.

It is worth noting that there are those who are fully accepting of the explanatory autonomy of reason-giving explanation, but who also claim that reasons are causes. For example (unlike, say, Anscombe, 1957 and the early Dennett, 1969) Davidson sees reasons as causes. Now, although I prefer not to think of reasons as causes, I am willing to accept for the sake of argument, that reasons are causes, especially if one accepts a counterfactual theory of causation. A counterfactual theory of causation is (roughly) one whereby A causes B, if and only if, had there not been A, there would not have been B. If I had not wanted to ask a question, I would not have raised my hand. In that very simple sense, my *desire* to ask a question was a cause of my *action*. However, it does not follow from something being a cause, that something is explanatory *in virtue of being a cause*. Since our explanatory concerns when we ask this special variety of “why” question are not causal concerns, the explanation given in terms of reasons is not a *causal explanation*, even if one thinks that reasons are causes. In short, conceding that reasons are causes does not concede that rational explanations (explanations in terms of reasons) are causal explanations.

So, what are our explanatory concerns when we ask for a personal explanation and why are they not causal? We want to understand the person *qua* agent, not *qua* causal system. Suppose somebody behaves in a way that, unlike a nervous tick, looks controlled and deliberate, and yet you still cannot give it an explanation. To take an unrealistic example, suppose someone holds up a poisonous mushroom, and announces: “This mushroom is deadly poisonous and I have no intention of killing myself” but then pops the mushroom in his mouth. This behavior is *surprising*, but it is not just surprise that you feel, but perplexity and confusion. In particular, you do not understand *why* this person ate the mushroom. You can hypothesize that he was lying, either about the poisonous nature of the mushroom, or his intentions to stay alive. Or he was demonstrating an antidote. Or he doesn't know the meaning of one or more of the words he was using. However, taken at face value, this action is perplexing. When this happens, we are not just bemoaning a failure to predict. There is more to it than this: we are perplexed by this *person qua agent*, by the fact that we find the person *unintelligible*. In fact, it seems that if his behavior can in no way be reinterpreted so as to confer intelligibility then the best way to understand it is as a brute causal process (perhaps he's a realistic-looking android, programmed to behave in just this way to prove the point I'm making here). But explaining it in these terms would require us to ask (and answer) a different question (“How come?” rather than “Why?”).

Other human beings often behave in ways that we have failed to anticipate, but that are still perfectly intelligible. We might say that, while causal and mechanistic explanations confer *predictability*, personal explanations confer *intelligibility*. Of course, assuming that people will be intelligible makes them more predictable, in the sense that it narrows down the ways they might behave in certain circumstances, but that does not mean that any given personal explanation improves, or is aimed at improving, our causal understanding of a person. Indeed, all of the causal understanding we need is already in place: we know that certain beliefs and desires give rise to certain actions, certain kinds of evidence give rise to certain beliefs. We just want to know, in this instance, what beliefs, or desires, or evidence the subject actually had, so that we can understand and evaluate them as persons.

## On the relationship between subpersonal and personal explanations

We have looked at what personal explanation is, and what causal and mechanistic subpersonal explanations are. But what is the relationship between them? We will start by looking at how they can compete, and then we will look at how they can inform each other.

### How they compete with each other

Personal and subpersonal explanations do not compete with each other in the way that they compete amongst themselves. Within a pragmatic framework competing explanations are competing answers to the *same question*. When you compare a personal and a subpersonal explanation, you are comparing answers to *different questions* (and indeed to different *kinds* of questions). However, competition comes in at a different level: at the level of asking the question in the first place. Asking a certain question presupposes that it is appropriate, that it can be answered, and that presupposes certain facts about the phenomenon in question. So, personal and subpersonal explanations will not directly compete (in the way that, as we are about to see, two personal explanations *can* directly compete). However, in some cases, both of them *being offered at all* will presuppose the obtaining of two incompatible states of affairs. Two demanders of explanations for a plane crash might ask: “Who was to blame for the crash?” or “What kind of weather conditions caused the crash?” Each implies different facts concerning the plane crash (e.g., the former implies that blame is to be attributed and that it wasn't a so-called “Act of God”). Asking a question betrays assumptions about the phenomenon that you are asking questions about. We will see more on this when we look at examples from delusion.

Another important way in which personal and subpersonal explanations compete is by imposing constraints on one another. If a personal explanation claims that, for example, the subject believed that *p* because they had an experience with a certain quality, but the best available subpersonal account suggests that the experience could not have been like that, then clearly these two explanations conflict. However, we will see that it is precisely through this constraining that the two kinds of explanation can inform each other.

### How they inform each other

We can use both personal and subpersonal explanations to further our understanding of the same individual. To use an example that will be relevant to us, we can ask about a patient with the Capgras delusion the subpersonal question, “How has this brain damage disrupted normal cognitive functioning?” A really good answer to this will make it altogether unmysterious why (how come) this particular damage has disrupted functioning in this particular way, and not in any other way. This will require causal and mechanistic explanations at different levels. However, one can also ask, “Why (on what grounds) does she believe what she does?” In answering this, you cannot use the same vocabulary as when answering the first, subpersonal, question. Dopamine dysregulation, modular damage, etc. none of these are even the right kind of thing to provide grounds for the subject. Similarly, to take a non-pathological case, you might ask me:

Q: Why did you think that James was at home?
And I might answer:
A: Because I saw his car in the driveway.

You would think it some kind of joke if I instead gave you a story about light hitting my retina, causing activation in V1, and so on. You want to know on what grounds I came to believe what I did.

Although subpersonal vocabulary (neurotransmitters, processing streams and so on) cannot feature directly in personal explanation, this is not to say that subpersonal psychology (broadly construed to include all the cognitive and brain sciences) cannot make very important contributions to personal explanations. In particular, it can make two very different kinds of contribution.

First, it can give us an idea of the nature of the grounds that a subject might have (e.g., what experiences or emotions they might be undergoing) and how it is that they have them, or rather, how come they have these experiences and not others. For example, as we are about to see, subpersonal psychology can suggest that the Capgras patient is experiencing a feeling of unfamiliarity toward a loved one. Once we understand what the subject may be experiencing, there is scope for their beliefs to be rendered *intelligible*, namely, to be subject to personal explanation. We can answer the question: “*Why* does the subject believe this?” In other words, the first kind of contribution that subpersonal psychology can make to personal explanation is one of suggesting the starting point for such an explanation.

The second kind of contribution that subpersonal psychology can make is very different. It may be able to warn us when personal explanation is *unavailable*. That is to say, it may warn us when any attempts at understanding the subject in terms of subjective grounds would be a waste of time. We saw with our plane crash analogy that an understanding of the situation may lead us to conclude that, for example, no blame is to be attributed. Similarly, an understanding of certain mentally ill individuals may lead us to realize that there is no answer to the “why” questions, “Why did he do this?,” “Why does he believe this?” Sometimes there may simply be *no grounds* for certain beliefs and behaviors, and it is vital that subpersonal psychology can warn us when this may be the case.

## An example from neuropsychiatry: delusion

Opposing views about the nature of delusional misidentification map on, in a nicely illustrative manner, to these two kinds of contributions that subpersonal psychology can make to personal explanation.

A key figure in the history of theoretical work on delusion is Karl Jaspers, who, in his *General Psychopathology* (1963), claimed that there were two very different projects: one of “understanding the subject,” and the other of rendering the psychopathological phenomenon causally tractable. The first project roughly corresponds to personal explanation, whereas the latter corresponds to subpersonal explanation. He thought that delusions (in particular the primary delusions of schizophrenia) arise without any grounds or justification; they are “un-understandable” in the sense that they are not intelligible. We cannot answer the question: “Why (viz. on what grounds) do these subjects believe what they do?” All we can do is try to understand the subject *qua* causal system.

However, half a century later the way was paved for (at least some delusions) to be rendered intelligible. In particular, Brendan Maher presciently hypothesized that the “delusional belief is not being held “in the face of evidence strong enough to destroy it,” but is being held because evidence is strong enough to support it” (1974, p. 99). What we then have to do as theorists is figure out what the evidence is, and how it arises. This will obviously have the potential to vary from one delusion to another, and may indeed provide a satisfying explanation of why some patients have some delusions and others have others (viz. there will be an un-mysterious connection between the nature of their experience, and the content of their delusion).

### The classic bottom-up model

This project received something of a breakthrough (a full 16 years later) in the case of the Capgras delusion (the delusion that one or more loved ones have been replaced by identical-looking impostors). Borrowing Bauer's (1984) model for facial processing, whereby there are two streams for processing facial information—one covert, affective and anatomically dorsal, the other overt, semantic, and anatomically ventral—Ellis and Young (1990) put forward the influential proposal that the Capgras delusion can be understood as a sort of “inverse prosopagnosia.”

People with prosopagnosia have difficulty in the overt recognition of faces. Show them a picture of a familiar face and they will not be able to tell you whose face it is. And yet, surprisingly, some of them appear to have differential autonomic responses (roughly, affective/emotional responses) to these faces, as measured by heightened skin conductance response (SCR). In other words, although they themselves cannot tell you whose face they are looking at, their affective system seems at the very least to be able to “tell” that it is someone familiar. Ellis and Young hypothesized that Bauer's two streams can be selectively impaired, leading to double dissociation. According to them, whereas with prosopagnosia the affective stream for “covert recognition” is intact and the semantic stream for “overt recognition” is impaired, with the Capgras delusion it is the other way around. This means that the Capgras patient is presented with someone who, thanks to intact semantic processing, looks to them exactly like a loved one, but there is a lack of affective response. The perceived person feels unfamiliar and the patient therefore concludes that this person cannot be the loved one in question. This model was given experimental support (Ellis et al., 1997) when it was discovered that, in contra-distinction to prosopagnosia, Capgras patients show diminished SCR when presented with familiar faces.

According to this etiology, there is scope for the Capgras delusion to be rendered intelligible, to be given a personal explanation, since it can be seen as something that is inferred on the basis of experiential evidence. These theories, which take delusions to be grounded in unusual experiences, are called “bottom-up” theories. A complete bottom-up account will contain a mix of personal and subpersonal explanation. The very existence of the anomalous experience is explained in terms of mechanism (in the Capgras case, on the Ellis and Young model, this could involve explaining how lesions disrupt affective processing of familiar faces). But the judgment itself is personal. The *person* infers from their experience. And the relevant question to ask is: “Why does the *person* believe that this woman is not his mother?” And the relevant answer is roughly: “Because this woman feels deeply unfamiliar to him.” This is not a causal, mechanistic explanation, but a personal one. And, if correct, it tells us all we need to know *within the scope of personal explanations*.

### Explanationist vs. endorsement accounts

However, there is a debate within bottom-up theories about what *precisely* the correct answer to this question (viz. “Why does this person believe that this woman is not his mother?”) is. Phrased in more technical terms, there is a debate about the content of the experience in the Capgras delusion. In other words, what exactly does the subject's experience tell her; how does it subjectively support her judgment?

To borrow Bayne and Pacherie's, (2004) terminology, “explanationist” accounts (e.g., Ellis and Young, 1990, Maher, 1999) claim that the content of the Capgras patient's experience is something *sparse* like, “This woman feels strange,” and that the delusional judgment *explains* the bizarre experience (roughly, the subject reasons: “This woman, in spite of looking like my mother, doesn't *feel* like my mother would feel, therefore she cannot *be* my mother”). The opposing accounts, so-called “endorsement” accounts (e.g., Bayne and Pacherie, 2004) claim that the delusional content is encoded directly in the unusual experience, and all that suffices is endorsement of that content. The content of the Capgras patient's experience, on such a view, is something *rich* like, “This woman is not my mother.”

Now, bracketing the plausibility of either account, it is worth noting an explanatory trade-off. As Pacherie (2009) points out, the explanationist account can more easily explain how the experience gets its content, since the content is so sparse. It can simply say that there is a disruption in emotional or affective processing. The task for subpersonal psychology is comparatively easy. However, it is a bigger explanatory step from the sparse content of the experience to the rich content of the delusion. One *prima facie* problem with this is that, if the experience is sparse and non-specific, why is there not a wider array of potential hypotheses used to explain it? (“Maybe I don't like mum anymore,” “Maybe I'm tired” etc.). In contrast, the endorsement theorist can get from the experience to the judgment just fine, since they have the same content. However, as Pacherie puts it, where “the endorsement account would appear to be weakest is in explaining how delusional patients could have the experiences that the account says they do” (Pacherie, 2009, p. 107). More precisely, subpersonal psychology needs to step in and tell us how it is that an experience can have a rich content like, “This woman is not my mother.”

Here we get a nice illustration of two directly competing personal explanations, namely, different answers to the very same “why” question. It also illustrates how these give rise to two different explanatory burdens that need to be picked up by subpersonal explanations. Presenting the competing accounts in terms of questions, where “Why?” and “How come?” questions correspond to the personal (justificatory) and subpersonal (mechanistic) questions respectively, we get (roughly) the following. For the explanationist account we get:

Q: “Why does the subject believe that his mother has been replaced by an impostor?”
A: “Because she feels unfamiliar to him.”
Q: “How come she feels unfamiliar to him?”
A: “Because affective processing has been disrupted in such and such a way.”

For the endorsement account we get:

Q: “Why does the subject believe that his mother has been replaced by an impostor?”
A: “Because his experience presents this woman as not being his mother.”
Q: “How come?”
A: “Because (for example) subpersonal mechanisms responsible for managing the representation of the identities of known individuals has been disrupted” (see e.g., Wilkinson, in press).

### Back to unintelligibility: top-down accounts

However, not everyone subscribes to bottom-up theories of delusions (Eilan, 2000; Campbell, 2001). In a way that harks back somewhat to Jaspers, these theorists claim that the delusion is not inferred, nor grounded in evidence, but caused. Any report (or even experimental evidence from SCR), for example, that the mother feels unfamiliar, is a consequence of (or an accompaniment to) the delusional belief, but not grounds for it. She feels unfamiliar because she is judged to not be the subject's mother, and not the other way around. As Campbell puts it, “‘delusion’ is a matter of top-down disturbance in some fundamental beliefs of the subject which may consequently affect experiences and actions” (2001, p. 89). An upshot of this is that the belief can only be explained subpersonally, and, of course, this leaves a large explanatory burden for subpersonal psychology. We cannot answer the question “*Why* does the *person* believe that this woman is not his mother?” We cannot appeal to grounds since there are none. We are back to Jaspers' claim that delusional subjects are “un-understandable.” The only question with an illuminating answer is: “What has *caused* this person to believe what she does?” This is precisely what I meant when I said that some etiologies will take personal explanation to not be available. However, note that, although, on these top-down theories, the delusional belief may not be amenable to personal explanation, any *action* performed on the basis of the belief will be, and this explanation will appeal to the belief. In such a situation we roughly get the following series of questions and answers.

Q: “Why did the patient stab her father (even though they seemed to have a good relationship prior to the event)?”
A: “Because she believed that he was not her father, but an identical-looking impostor.”
Q: “And on what grounds did she believe this?”
A: “There were none. The belief was merely caused.”

At this point we would need to delve into the subpersonal mechanisms to understand what is underpinning the (groundless) belief in terms of mechanisms.

## An illustrative objection

Somebody might say that the “kinds of explanation” constraint is illusory, and, in particular, that personal explanation has no place in a scientific enterprise. When people believe things on epistemic grounds, or they do things for reasons, nothing fundamentally different is going on. This could, in principle, all be explained subpersonally by the cognitive sciences.

I think this is a misleading way to think, and I would like to run through a thought experiment to illustrate why. Suppose we had a “complete” subpersonal description (whatever that means!) of what is going on, say, in an instance of thought insertion. We are not appealing to anything personal, we are not talking about grounds or reasons, just mechanistic stuff. Assuming physicalism, and highly advanced imaging techniques at our disposal, we could, in principle, take any given individual and see when they would (and when they would not) report inserted thoughts. I am very happy to grant this. Thought insertion is, in a rather trivial sense, a fundamentally physical phenomenon. However, suppose that it happens that thought insertion, the denial of ownership of one's thoughts, is actually grounded in a very bizarre experience. We *could* know exactly what is going on in the brain of someone (i.e., we know what that neural activity looks like on the scanner) who is reporting inserted thoughts, but still not know on what grounds they are denying ownership of their thoughts (or indeed that there are grounds at all). This seems like a possible epistemic state for us, as scientists, to be in. More worryingly, it entails ignorance of an important and irreducible fact (by “fact,” I mean, a claim that is determinately true) namely, concerning the grounds on which somebody is denying ownership of her thoughts.

As the section in this paper that examines the relationship between personal and subpersonal explanations makes clear, I am not denying that we could work out, to some extent, the subject's experiential grounds from subpersonal data. Here, I have advocated the fruitful, but careful, contribution of subpersonal psychology to personal explanation. But this in itself requires us to take personal explanation seriously. In this thought experiment, personal explanation is disregarded, eliminated, out of bounds. And the intuition I hope you share is that this entails a kind of ignorance.

Of course, where this illustrative objection goes awry is in making the fallacious step from physicalism to reductionism. Everything in the universe could well be made of physical stuff, but as human beings we are constrained, partly by our interests and concerns, and partly by our cognitive and conceptual limitations. We therefore talk about, and explain, many different things (and kinds of things) in many different ways (and kinds of ways). Obviously, there are facts concerning people that go beyond, and are not reducible to, neural facts. There are epistemic facts (facts about grounds for belief), motivational facts (facts about people doing things for reasons). There are also facts about what people experience (it is, for example, a fact, as unambiguously true as anything, that I am currently not in excruciating pain). And it does not stop there: there are social facts, economic facts, contractual facts, and so on. You cannot capture what it is for me to sign a contract using physics and physiology.

## Summary and conclusion

In this paper, I built on a pragmatic view of explanation, on the basis of which explanations are answers to explanation-demanding questions, in order to show a distinction between levels of explanation and kinds of explanation. Different kinds of questions, and hence explanations, arise because we have different kinds of explanatory concerns. One important kind of explanatory concern involves asking a certain justificatory kind of “why” question. Answering this kind of question provides a personal explanation, namely, renders intelligible the beliefs and actions of other persons. I then used contrasting theories of delusional misidentification to illustrate how different facts about the phenomenon that is being explained impose (i) constraints on the availability of personal explanation and (ii) leave different explanatory burdens for subpersonal psychology (broadly construed). More generally, this also illustrated how asking certain kinds of questions, seeking certain kinds of explanations, carries implicit assumptions about the nature of the phenomenon about which the questions are being asked.

### Conflict of interest statement

The author declares that the research was conducted in the absence of any commercial or financial relationships that could be construed as a potential conflict of interest.
